# Cognitive Implications of White Matter Alterations in Overweight Chinese Individuals: An NODDI and DTI Study

**DOI:** 10.1002/brb3.70777

**Published:** 2025-08-22

**Authors:** Shaofan Jiang, Yingzhe Cheng, Rifeng Jiang, Peilin Huang, Jiejun Zhang, Xiaodong Pan, Yunjing Xue

**Affiliations:** ^1^ Department of Radiology Fujian Medical University Union Hospital Fuzhou China; ^2^ Department of Neurology, Center for Cognitive Neurology Fujian Medical University Union Hospital Fuzhou China; ^3^ Fujian Institute of Geriatrics Fujian Medical University Union Hospital Fuzhou China; ^4^ Institute of Clinical Neurology Fujian Medical University Fuzhou China; ^5^ Fujian Key Laboratory of Molecular Neurology Fujian Medical University Fuzhou China; ^6^ Center For Geriatrics Hainan General Hospital Hainan China; ^7^ Department of Clinical Nutrition Shandong Provincial Hospital Affiliated to Shandong First Medical University Jinan Shandong China

**Keywords:** cognition, MRI, overweight, plasma biomarker

## Abstract

**Objective:**

Scarce studies have elucidated the relationship between the microstructure changes of white matter (WM) and cognitive domains in overweight (OW). This study aimed to evaluate the WM microstructure changes and the relationship with the cognitive domains and cognition‐related plasma biomarkers.

**Methods:**

This study recruited 77 standard weight (SW) and 42 OW participants. The WM integrity was analyzed using neurite orientation dispersion and density imaging (NODDI) and diffusion tensor imaging (DTI). The region of interest (ROI) values of NODDI and DTI were calculated and compared between groups. The correlations between WM microstructure and cognitive domains and cognition‐related plasma biomarkers were also recorded.

**Results:**

The ROI analysis revealed significant differences in fractional anisotropy (FA) and orientation dispersion index (ODI) in extensive WM regions (*p* < 0.05). Further analysis showed that NODDI parameter values of the uncinate fasciculus (UF) showed significant correlations with the cognitive domain. Additionally, other abnormal WM microstructures of OW were associated with cognitive domains and cognition‐related plasma biomarkers to varying degrees.

**Conclusion:**

The findings evidence that abnormalities of the WM microstructure may indicate typical changes of OW, and these microstructure abnormalities are associated with cognitive functions and cognition‐related plasma biomarkers.

## Graphical abstract

The abnormal white matter (WM) microstructure was localized to the WM fiber bundles in overweight (OW) participants. Moreover, these abnormal WM microstructures are associated with broad cognitive domains and cognition‐related plasma biomarkers. The findings may provide novel insights into the impact of abnormal WM microstructure on the cognition of OW, suggesting the necessity of early intervention in clinical practice.

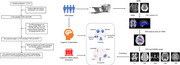

## 1 Introduction

Dementia is expected to affect 153 million people by 2050 (Nichols et al. 2022). Several risk factors are associated with an increased risk of cognitive impairment, including body mass index (BMI) (Alhurani et al. 2016). Previous studies have shown that underweight (Ren et al. 2021) and obesity may confer a risk for cognitive impairment (Qu et al. 2020, Kivimäki et al., 2017), and their close correlation with the integrity of white matter (WM) microstructure based on diffusion tensor imaging (DTI) (Fessel, 2022, He et al. 2015). Increasing attention has been paid to associations between weight status, measured by BMI, and cognitive function in older populations. However, there has been controversy about the relationship between overweight individuals and their connection to the broad cognitive domain. Some research suggests that higher BMI contributes to poor cognitive function (Liang et al., 2022, Manacharoen et al., 2023). Nevertheless, another recent study has revealed that being overweight is not associated with cognitive decline (O'Donovan et al., 2022). Although there is no consensus, it has been widely proposed that unfavorable weight status may affect metabolic functions and promote inflammation, which could increase the risk of poor cognitive function (Grillner et al., 2016). Changes in cognitive function are related to many biological factors. Among them, plasma biomarkers, as an important tool for assessing cognitive health, have been widely used. Cognition‐related plasma biomarkers are usually indicators of biological processes that reflect brain health and function, such as β‐amyloid (Aβ), glial fibrillary acidic protein (GFAP), neurofilament light chain (NfL), and phospho‐tau181 (P‐Tau181) (Lee et al., 2019). Plasma amyloid β‐42 (Aβ42) and amyloid β‐40 (Aβ40) are two major isoforms of amyloid beta peptides, which are key biomarkers for Alzheimer's disease and other neurodegenerative conditions (Gentreau et al., 2022). NfL is an axonal protein that is released when peripheral neurons are damaged, subsequently leading to an increase in NfL concentration (Huehnchen et al., 2022). GFAP is a type III intermediate filament protein that is primarily expressed in astrocytes of the central nervous system. (Huang et al., 2023). NfL, GFAP, and phosphorylated tau at threonine 181 (P‐tau181) are brain‐derived molecules that, when detected in the serum or plasma, are thought to indicate ongoing inflammation and damage in the central nervous system (Zuroff et al., 2022). Therefore, studying the relationship between these plasma biomarkers and cognitive function in overweight individuals can help understand how being overweight affects brain structure and function through these biomarkers.

DTI, as a classical diffusion imaging method, which summarizes the overall diffusion in a voxel with a simple tensor, is widely used to diagnose diseases of the central nervous system, including tumors, cognitive impairment, epilepsy, and multiple sclerosis (Hu et al., 2022, Wang et al., 2022). The advantage of neurite orientation dispersion and density imaging (NODDI) is that by explicitly modeling multiple compartments (intra‐ and extra‐neurite, and free water) that can contribute to the observed diffusivity of the voxel, it allows more specific inferences about the microstructural properties beyond overall diffusivity. By taking into account the orientation and density of neurons, NODDI provides more sensitive measures of neuronal health and integrity, making it a powerful tool for studying white matter in both healthy and diseased states (Palacios et al., 2020). DTI and NODDI reveal abnormal changes in WM and improved interpretability in clinical imaging of the human brain in terms of diffusion‐weighted microstructural features.

Although increasing attention has been paid to the relationship between being overweight and brain health in recent years (Rodriguez‐Ayllon et al., 2020, Stillman et al., 2022), studies specifically using advanced imaging techniques (such as NODDI) to investigate the impact of being overweight on white matter microstructure are still relatively scarce. A recent study suggested that high cumulative BMI was associated with smaller brain volume, larger volume of white matter lesions, and abnormal microstructural integrity (Lv et al., 2024). Some literature has shown that, compared with normal‐weight children, obese children exhibit differences in white matter organization, mainly in the frontal and temporal lobe regions (Ou et al., 2015) and corpus callosum (Rodriguez‐Ayllon et al., 2024). However, no relevant studies have been conducted in elderly populations. This knowledge gap provides an important opportunity for further exploration. We hypothesize that individuals who are overweight may exhibit changes in white matter integrity, which may be associated with cognitive function and cognitive‐related plasma biomarkers.

Therefore, this study employed DTI and NODDI to analyze the white matter microstructure in overweight adults. We further explored the relationship between white matter abnormalities, cognitive function, and plasma biomarkers related to cognition. These findings may provide new insights into the neural mechanisms underlying cognitive decline associated with overweight and support the potential use of advanced neuroimaging techniques as an early assessment tool.

## 2 Methods

### 2.1 Ethical Approval Statement

All procedures performed in studies involving human participants followed the ethical standards of the institutional research committee and the 1964 Helsinki Declaration and its later amendments or comparable ethical standards. The study protocol was approved by the Ethics Committee of Fujian Medical University Union Hospital(2021KJT001).

### 2.2 Study Subjects

Participants in this study were consecutively recruited from the Cognitive Psychology Outpatient Department of Fujian Union Hospital between May 2023 and February 2024. Participants voluntarily sought cognitive testing with concerns about their own cognitive functions. The inclusion criteria required that participants have healthy metabolism with no significant cognitive deficits on standardized tests and clinical assessments. Exclusion criteria encompassed obesity, underweight, other neurological diseases, mental illness, severe endocrine disorders, injury or surgery within the past 6 months, use of illegal anesthetics or alcohol abuse, pregnancy, use of psychiatric drugs, anesthetic analgesics, benzodiazepines, psychoactive substances, and inability to cooperate or cognition test.

All participants underwent a magnetic resonance imaging (MRI) examination on the day they completed the cognitive assessment. Demographic characteristics were collected, including age, sex, education, weight, and height. BMI was calculated using the formula BMI  =  (weight in kg)/(height in meters)^2^. BMI is a key indicator of underweight/ standard weight (SW)/overweight (OW)/obesity (Liu et al., 2023), and the World Health Organization (WHO) has developed an international standard, in which the normal range of BMI is 18.5–24.9 kg/m^2^, and a BMI of 25–29.9 kg/m^2^ as overweight. However, the enrolled participants in our study were all Chinese people, the WHO's BMI standard is not applicable (Liu et al., 2023). Therefore, the current study adopted the reference standard for adult BMI in China, that is, 18.5–23.9 kg/m^2^ as SW, and 24–27.9 kg/m^2^ as OW.

**FIGURE 1:**
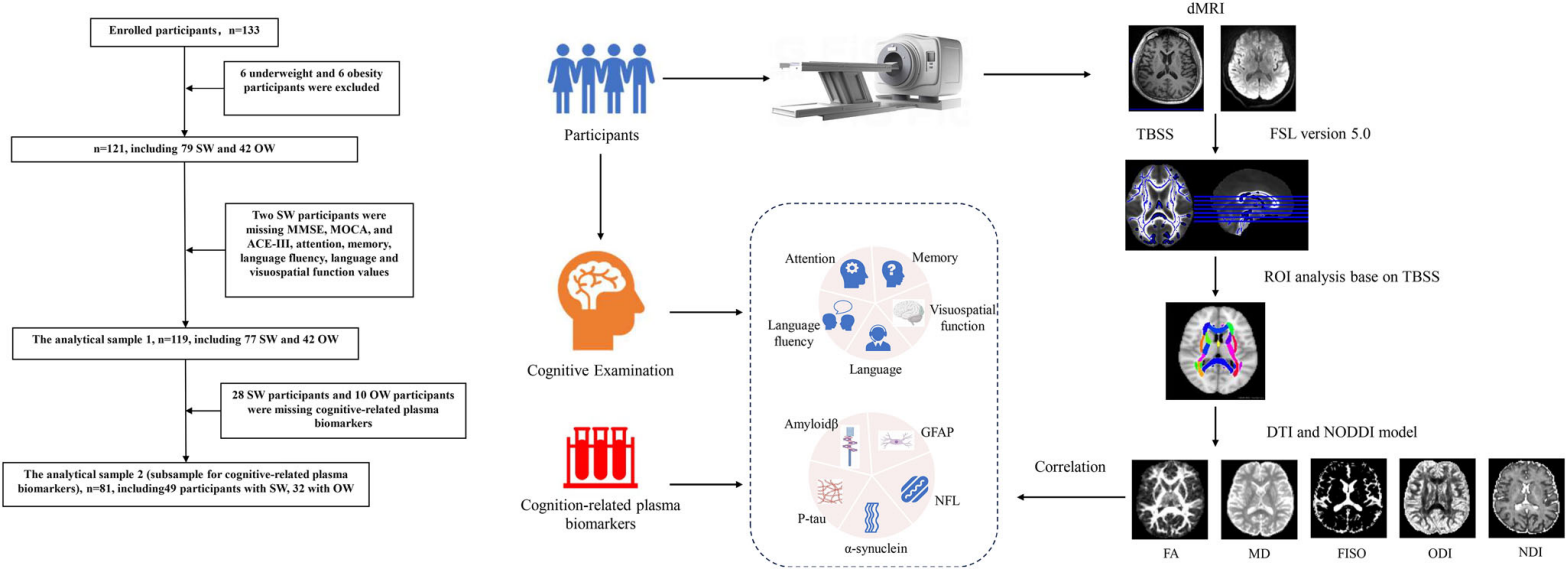
The enrollment process and flowchart for the participant. MMSE, mini‐mental state examination; MOCA, Montreal Cognitive Assessment; ACE‐III, Addenbrooke's Cognitive Examination version III; SW, standard weight; OW, overweight.

Figure 1 shows the flowchart of the study. The biomarkers, demographic characteristics, and the assessment of cognitive function were all collected on the same day as the MRI scan to ensure temporal consistency of the data.

### 2.3 Assessment of Cognitive Function

Global cognition, including mini‐mental state examination (MMSE) (Katzman et al., 1988), Montreal Cognitive Assessment (MOCA) (Lu et al., 2011), Rey Complex Figure Test‐Immediate recall (RCFT‐I), Rey Complex Figure Test‐Delayed recall (RCFT‐D) (Le Osterrieth, 1944), and Addenbrooke's Cognitive Examination version III (ACE‐III) scales (Wang et al., 2017), was conducted by a neurologist with 5 years of clinical experience. Cognitive function in five subdomains, including attention, memory, language, language fluency, and visuospatial function, was also conducted (Wang et al., 2017). Having been extensively validated, this cognitive function battery is effective in evaluating different aspects of cognitive function (Cheng et al., 2024, Lee et al., 2023).

### 2.4 Determination of Plasma Biomarkers

Blood samples were collected into EDTA‐coated vacuum containers. After centrifugation, blood samples were stored at −80°C and thawed immediately before composition quantification. The levels of plasma Aβ42, Aβ40, GFAP, NfL, phospho‐tau181 (P‐tau), and α‐synuclein were measured using the human Neurology 3‐Plex A Assay (N3PA) kit on an automatic single‐molecule‐array (SIMOA) instrument (Quanterix Corp, MA, USA) according to the manufacturer's protocol.

### 2.5 MRI Data Acquisition

All the images were acquired using a 3T MR scanner (MAGNETOM Prisma, Siemens Healthcare) with a 64‐channel head coil. The foam was placed around the head of each participant before the test to reduce head movement. All participants were asked to refrain from moving their heads during the MRI scan. Diffusion‐weighted images were obtained by using a multi‐shell echo‐planar imaging sequence, which consisted of four *b*‐values (0, 1000, 2000, and 3000 s/mm^2^) along 6, 30, 30, and 30 gradient directions, respectively; 72 slices with a thickness of 2 mm were used. The other scan parameters were as follows: repetition time (TR), 5800 ms; time to echo (TE), 91 ms; field‐of‐view (FOV), 215 × 215 mm^2^; GeneRalized Autocalibrating Partial Parallel Acquisition, 2; slice acceleration factor, 2; number of averages, 1; voxel size, 2×2×2 mm^3^, without gap; and acquisition time, 9 min 44 s.

### 2.6 MRI Data Postprocessing

In this study, DTI and NODDI were both employed to assess white matter. All images were scrutinized and any affected by artifacts, noise, or head movement were excluded. The remaining images were processed into raw, preprocessed files with specific indices. (1) The initial DICOM format images were converted into NIFTI files using the MRIcron software package (http://www.nitrc.org/projects/mricron). (2) An average *b* = 0 s/mm^2^ image from each diffusion‐weighted shell served as a reference for subsequent steps. (3) We performed motion and eddy current correction using FSL's eddy tool in the FMRIB's Diffusion Toolbox (FDT). (4) Brain mask generation using a brain extraction tool (BET) and distortion correction via registration of individual dMRI data. (5) We used visual inspection to avoid severe geometric distortions, ensuring that all images met the quality control criteria before further analysis. (6) The FMRIB's Diffusion Toolbox (FSL‐FDT, included in FSL version 5.0, (http://fsl.fmrib.ox.ac.uk/fsl/fslwiki/FDT) was utilized to remove the skull from the images. Following this, the DTI parameters (including FA and MD) and NODDI parameters (including NDI, ODI, and FISO) were calculated with NODDI_toolbox (www.nitrc.org/projects/noddi_toolbox) in MatLab R2022b (The MathWorks Inc., Natick, MA, USA).

### 2.7 TBSS and ROI Analysis

Tract‐based spatial statistics (TBSS, part of FSL version 5.0, http://fsl.fmrib.ox.ac.uk/fsl/fslwiki/TBSS/UserGuide) was conducted with the FSL software. In our TBSS analysis pipeline, the skeleton projection of MD, NDI, ODI, and FISO was based on the FA skeleton, which is consistent with the standard TBSS method. The main steps were as follows: (1) All individual FA images were nonlinearly registered to the FMRIB58_FA template using FNIRT (FMRIB's Nonlinear Registration Tool). The FMRIB58_FA template is already in Montreal Neurological Institute (MNI) 152 standard space. (2) An average FA image was created from continuous scan images of all subjects in this common space and refined to generate an average WM skeleton representing the center of all fiber bundles with a threshold greater than 0.2. (3) Each subject's FA data were then projected onto a mean white matter skeleton by assigning each skeleton voxel the highest FA value from the local perpendicular tract, ensuring alignment across subjects. The MD, NDI, ODI, and FISO images were subjected to the same nonlinear deformations at the individual level and projected onto the FA skeleton using nearest‐neighbor projection. This ensures that the projection points for all indices are consistent with the voxels of the FA skeleton. Therefore, the values of MD, NDI, ODI, and FISO on the skeleton originated from the same spatial locations, avoiding the situation where projection points for different indices come from different voxels. (4) The FSL was used to conduct a double independent sample *t*‐test and a replacement test between the two groups (replacement times 5000 times), and the differences in the MD, NDI, ODI, and FISO values of the WM between the two groups were detected. After the clustering localization based on TBSS analysis, we further extracted the NODDI parameters of the key regions of interest (ROIs) related to cognition (Ou et al., 2015, Rodriguez‐Ayllon et al., 2024). The ROIs from Johns Hopkins University ICBM‐DTI‐81 White‐Matter Labels (Liu et al., 2020) were used in this analysis. The image metrics for NODDI were transformed to MNI152 space, and then the mean values of FA, MD, FISO, NDI, and ODI were extracted from each tract. Age and sex were added as covariates.

To explore the association between abnormal white matter microstructure and cognition, we performed correlation analyses between extracted DTI/NODDI metrics from selected ROIs and cognitive performance scores (MMSE, MOCA, ACE‐III, RCFT‐I, RCFT‐D, and cognitive subdomains), as well as plasma biomarker levels (including Aβ42, Aβ40, GFAP, NfL, p‐tau181, and α‐synuclein). Age, sex, and education level were included as covariates. The false discovery rate (FDR) correction was applied to control for multiple comparisons.

### 2.8 Statistical Analysis

All statistical analyses were performed using SPSS (Version 26.0; Chicago, Illinois, USA). All statistical analyses were defined as two‐sided *p* < 0.05. Data were tested for normal distribution by the Kolmogorov–Smirnov test. Continuous variables were expressed as the mean ± standard deviation by *t*‐test. The non‐normal distribution variables were expressed as median (interquartile range, IQR) and compared by a non‐parametric test. The chi‐squared and Fisher's exact tests were used to compare categorical variables. Finally, Pearson's partial correlation analysis (controlling for sex, age, and total years of education) was utilized to determine associations between abnormal WMs and plasma biomarkers, and cognitive function. The statistical significance threshold was set at *p* < 0.05.

## 3 Results

### 3.1 Demographic Characteristics of Participants

One hundred and nineteen participants were enrolled. 28 participants were missing plasma‐based markers in the SW group, and 10 participants were missing plasma‐based markers in the OW group (Table 1). There were no significant differences in age, sex, education, diabetes, hyperlipidemia, hypertension, MMSE, MOCA, ACE‐III, RCFT‐I, RCFT‐D, CDR, attention, memory, language fluency, language, and visuospatial function (*p* > 0.05), but there were significant differences in height, weight, and BMI between the two groups (*p* < 0.01). 28 participants were missing plasma‐based markers in the SW group, and 10 participants were missing plasma‐based markers in the OW group. There were no significant differences in Aβ42, Aβ40, Aβ42/ Aβ40, P‐tau, and α‐synuclein (*p* > 0.05), but there were significant differences in GFAP and NfL between the two groups (*p* < 0.05).

**TABLE 1:** Characteristics of the study participants.

Characteristics	SW (*n*=77)	OW (*n*=42)	*F*/*Z* or χ2 value	*p* value
Age	61.21 ± 12.91	62.05 ± 10.81	1.746	0.721
Female (*n*, %)	43 (55.8%)	17(38.6%)	2.568	0.109
Education(years)	10.00 ± 4.99	10.60 ± 5.62	0.311	0.553
MMSE	28.00 (7.00)	28.50 (4.00)	−0.754	0.451
MOCA	27.00 (10.00)	25.00 (8.00)	−0.900	0.368
ACE‐III	85.00 (27.00)	85.00 (17.00)	−0.501	0.616
RCFT‐I	33.00 (13.00)	32.50 (13.00)	−0.062	0.951
RCFT‐D	7.00 (13.00)	7.00 (11.00)	−0.042	0.967
CDR	0.50 (1.00)	0.50 (1.00)	−0.246	0.805
Attention	18.00 (4.00)	18.00 (1.00)	−1.789	0.074
Memory	20.00 (14.00)	20.50 (8.00)	−0.201	0.841
Language fluency	8.38 ± 3.43	9.57 ± 3.95	0.038	0.088
Language	24.00 (6.00)	25.00 (3.00)	−1.472	0.141
Visuospatial function	15.00 (4.00)	15.00 (3.00)	−1.003	0.316
Height (cm)	161.77 ± 7.61	165.73 ± 7.73	0.237	0.008^**^
Weight (KG)	56.05 ± 7.25	70.25 ± 7.30	0.002	<0.001^***^
BMI (kg/m^2^)	21.35 ± 1.52	25.52 ± 0.94	13.033	<0.001^***^
Aβ42^a^	6.63 ± 1.69	6.60 ± 1.36	0.740	0.927
Aβ40 ^a^	120.60 ± 28.45	120.36 ± 25.69	0.417	0.969
Aβ42/ Aβ40 ^a^	0.06 ± 0.01	0.06 ± 0.01	1.284	0.995
P‐tau ^a^	2.36 (1.73)	1.77 (1.01)	−1.584	0.113
GFAP ^a^	90.61 (91.20)	76.49 (35.77)	−2.164	0.030^*^
NfL ^a^	24.17 ± 15.76	16.94 ± 10.34	5.049	0.015^*^
α‐synuclein ^a^	12362.57 (30048.67)	8406.81 (15786.40)	−0.628	0.530

*Note*: Data are presented as number (percentage), median (IQR).

Abbreviations: ACE‐III, Addenbrooke's Cognitive Examination version III; Aβ40, Amyloidβ‐40; CDR, Clinical Dementia Rating. Aβ42, Amyloidβ‐42; GFAP, glial fibrillary acidic protein; MMSE, mini‐mental state examination; MOCA, Montreal Cognitive Assessment; NfL, neurofilament light chain; OW, overweight; P‐tau, phospho‐tau181; RCFT‐D, Rey Complex Figure Test‐Delayed recall; RCFT‐I, Rey Complex Figure Test‐Immediate recall; SW, standard weight.

^a^28 participants were missing plasma‐based markers in the SW group; 10 participants were missing plasma‐based markers in the OW group.

***p < 0.001, ^**^ *p* < 0.01, ^*^ *p* < 0.05.

### 3.2 Results of TBSS Analysis

**FIGURE 2:**
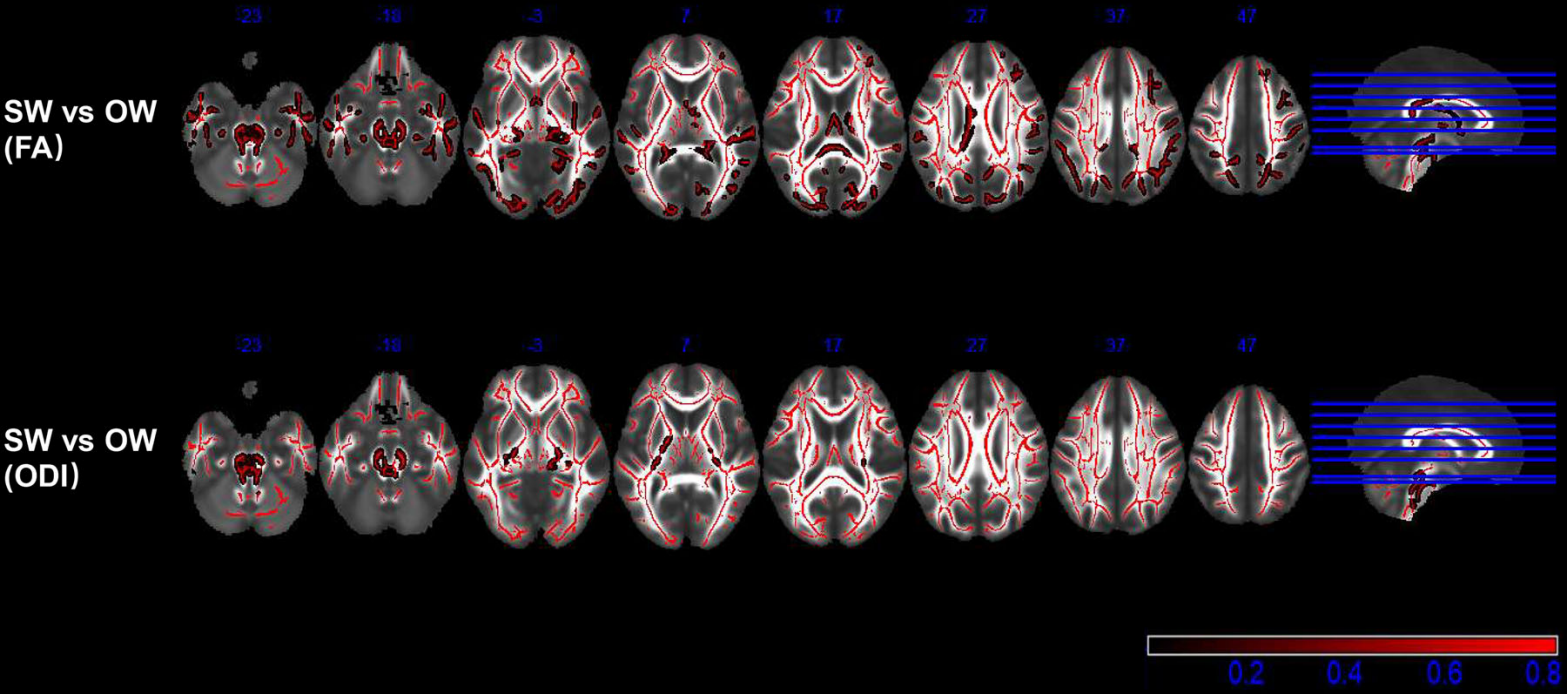
The results of diffusion metrics by tract‐based spatial statistics (TBSS). SW, standard weight; OW, overweight; ODI, orientation dispersion index; FA, fractional anisotropy. Supra‐threshold voxels have been enlarged using the TBSS fill (part of FSL) for illustrative purposes. Gray, the mean FA value; red, the average skeleton; bold red shows significant differences (permutation tests, *p* < 0.05, FDR corrected).

TBSS statistical analysis showed significant differences in FA and ODI in several cerebral regions between SW and OW groups, including middle cerebellar peduncle (MCP), right and left cerebral peduncle (CP), right and left inferior fronto‐occipital fasciculus (IFOF), right and left UF in FA and MCP, right and left CP in ODI, which were hereinafter referred to as comprehensive abnormal fiber bundles (*p* < 0.05). In addition, no significant differences in MD, NDI, and FISO were found between the SW and OW groups (*p* > 0.05; Figure 2 and Table 2).

**TABLE 2:** Anatomical regions of tract‐based spatial statistics results (SW vs. OW).

NOH vs. SOH	Cluster index	Voxels	*p*	Anatomical regions (Top 3)	MAX X (mm)	MAX Y (mm)	MAX Z (mm)
ODI	1	3787	0.008	Middle cerebellar peduncle:14.7679 Cerebral peduncle R:10.9705 Cerebral peduncle L:9.0717	14	−23	−32
FA	7	14814	0.014	Inferior fronto‐occipital fasciculus R:1.7694 Uncinate fasciculus R:0.6667	35	−62	40
	6	7766	0.031	Inferior fronto‐occipital fasciculus L:1.1853 Uncinate fasciculus L:0.4310	−9	−47	44
	5	5883	0.026	Cerebral peduncle R:8.3916 Cerebral peduncle L:6.4336 Middle cerebellar peduncle:3.4965	9	−18	−28

Abbreviations: MD, mean diffusivity; ODI, orientation dispersion index; OW, overweight; SW, standard weight.

### 3.3 ROI Analysis Results

**FIGURE 3:**
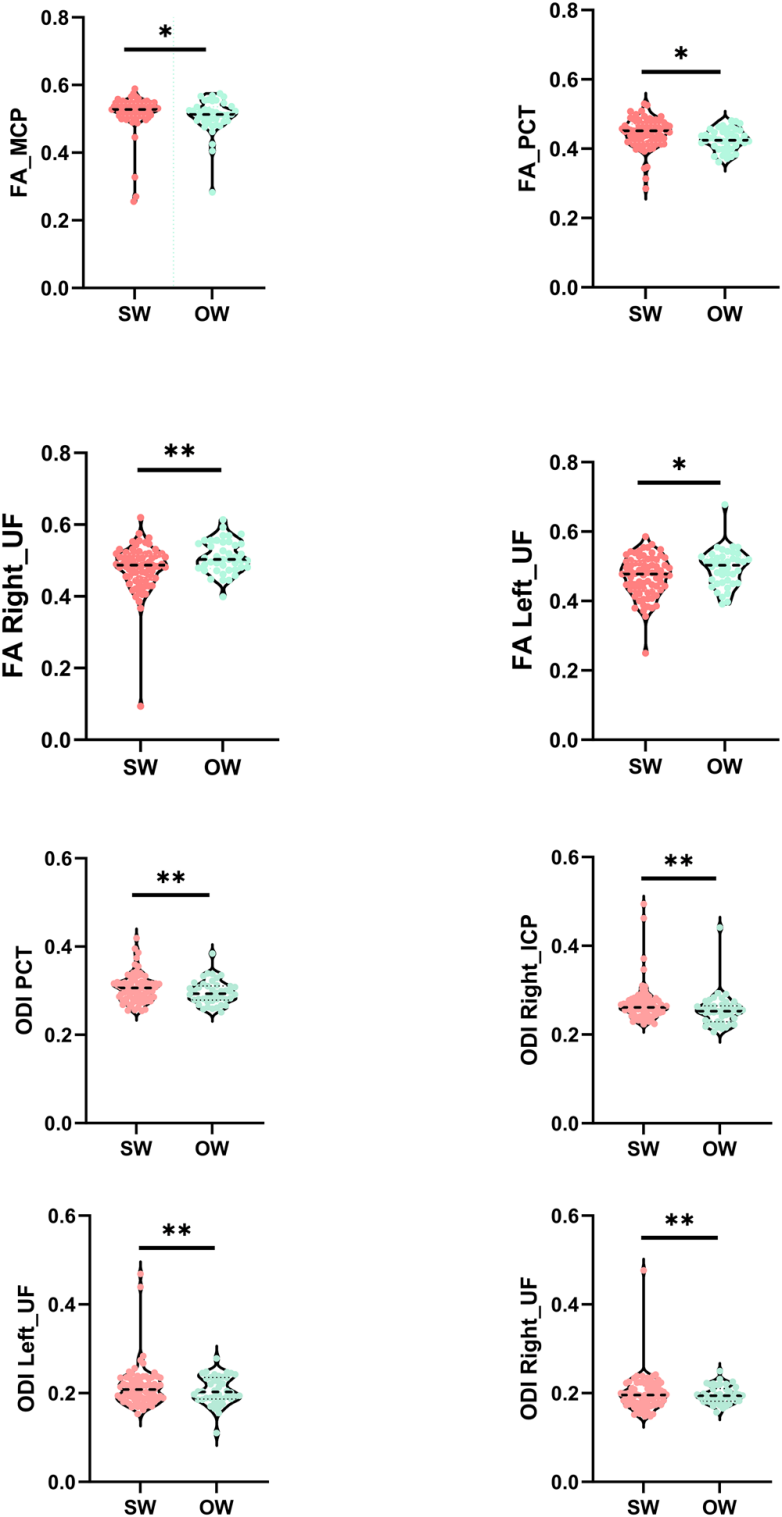
Violin plots (A) show the important fiber bundles of FA in the region of interest (ROI) by tract‐based spatial statistics (TBSS). Violin plots (B) show the important fiber bundles of ODI in the ROI by tract‐based spatial statistics (TBSS). SW, standard weight; OW, overweight; FA, fractional anisotropy; ODI, orientation dispersion index. *p <  0.05; **p <  0.01; ***p <  0.001.

Compared with the SW group, FAs of the pontine crossing tract (PCT) and MCP in the OW group increased to different degrees; FAs of bilateral UF in the OW group decreased to varying degrees; ODIs of PCT, right ICP, and bilateral UF in the OW group decreased to varying degrees (*p* < 0.05, FDR‐corrected; Figure 3). In addition, no significant differences in other fiber bundle indicators were observed between the groups (*p* > 0.05).

### 3.4 Association of Aberrant DTI and NODDI Indicators With Cognition

**FIGURE 4:**
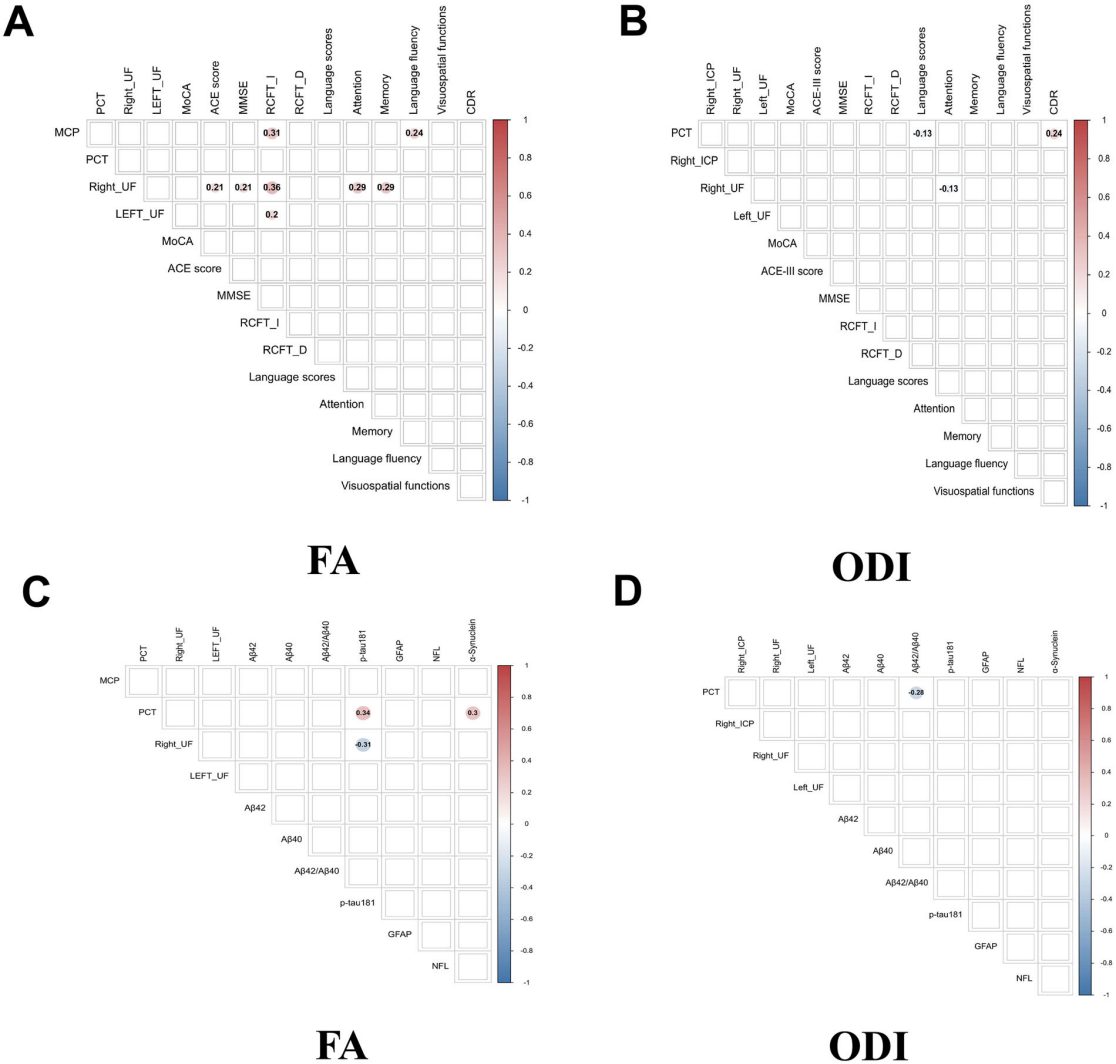
The partial correlation results between diffusion metrics with cognitive function and cognition‐related biomarkers. (A) Correlation between the FA of abnormal fiber bundles and cognitive function. (B) Correlation between the ODI of abnormal fiber bundles and cognitive function. (C) Correlation between the FA of abnormal fiber bundles and cognition‐related plasma biomarkers. (D) Correlation between the ODI of abnormal fiber bundles and cognition‐related plasma biomarkers. FA, fractional anisotropy; ODI, orientation dispersion index.

The FA of the MCP was positively associated with RCFT‐I and language fluency. The FA of the right UF was positively associated with ACE‐III, MMSE, RCFT‐I, attention, and memory. The FA of the left UF was positively associated with RCFT‐I (*p* < 0.05; Figure 4A).

The ODI of the PCT was negatively associated with language and positively associated with CDR. The ODI of the right UF was negatively associated with attention (*p* < 0.05; Figure 4B).

Furthermore, no association was found between other fiber bundle indexes and cognitive function (*p* > 0.05; Figure 4A,B).

### 3.5 Association of Aberrant DTI and NODDI Indicators With Cognition‐Related Plasma Biomarkers

The FA of the PCT was positively associated with α‐synuclein and p‐tau181. The FA of the right UF was negatively associated with p‐tau181 (*p* < 0.05; Figure 4C). The ODI of the PCT was negatively positively associated with Aβ42/Aβ40 (*p* < 0.05; Figure 4D). No association was observed between fiber bundle indexes of FA/ODI and cognition‐related plasma biomarkers (*p* > 0.05; Figure 4C,D).

## 4 Discussion

In this DTI and NODDI study, we utilized TBSS‐based ROI to analyze variations in the integrity and microstructure of WM in participants with OW and SW. We found that participants in the OW group exhibited WM discrepancies in various regions and severities, and these microstructure changes were associated with cognitive function and cognition‐related plasma biomarkers to varying degrees. Taken together, these findings indicate that microstructural abnormalities in WM may be indicative of underlying histopathological changes linked to early cognitive decline, and the compromised integrity of these fiber bundles in OW participants may serve as a potential marker. These findings may shed light on the effects of OW on cognitive domains.

We used the DTI and NODDI models sensitive to WM microstructure to assess WM, especially the NODDI model. DTI uses two parameters: FA and MD, while NODDI uses three parameters: NDI, ODI, and FISO, each with a scalar value ranging from 0 to 1. The FA values were most frequently used to estimate diffusion anisotropy, and the MD values were considered to clearly reflect the molecular mean diffusivity under restricted motion (Liu et al., 2020). NDI estimates the volume fraction of neurite, which is related to neurite density and myelin density, ODI estimates the dispersion index of neurite which was a stronger correlation with the directional dispersion of histological measurements than FA (Zhang et al., 2012), while FISO reflects the content of free water in tissue, which is used to quantify the volume of voxels occupied by free‐flowing cerebrospinal fluid (CSF) (Anaby et al., 2022). Cumulative studies have shown that the decrease in FA and ODI, and the increase in MD, NDI, and FISO may indicate a decrease in the microstructural integrity of the WM (Palacios et al., 2020, Liu et al., 2020, Fu et al., 2019). However, in this study, the increases in FAs of bilateral UF are inconsistent with previous reports (Faraji et al., 2023), which may result from the selective retention (or selective denaturation) of specific fibers in areas where fibers cross and disperse, increasing the arrangement of the remaining fiber bundles (Hu et al., 2022).

Most of the previous OW studies have focused on changes in gray matter (Gupta et al., 2015) or the integrity of the WM based on DTI (Carbine et al., 2019, Xu et al., 2011), and less research involving the microstructure based on NODDI. In this study, we captured several microstructural anomalies in the WM of the OW group, especially in ODI, which implies that the fiber bundles of the WM are likely to undergo injury, necrosis, and demyelination of nerve fibers. It is worth noting that an abnormality was present in the levels of FA and ODI in the bilateral UF and PCT (part of MCP), which may indicate typical changes of the WM in OW patients. In this study, we found several significant anomalies in FAs of MCP, PCT, and bilateral UF in the OW group, while the ODI of OW showed more regional microstructure abnormalities. ODI appears to be more sensitive to axonal degeneration than FA.

Previous studies found that loss of white matter integrity was associated with BMI, particularly in the area connecting the temporal and frontal lobes (Kullmann et al., 2015). Obese adults have poorer WM integrity in UF (Kullmann et al., 2015, Karlsson et al., 2013), cerebral peduncle (Repple et al., 2018), corona radiata (Zhang et al., 2018), corticospinal tract (Carbine et al., 2019, Xu et al., 2011), cingulu (Park et al., 2019), and corpus callosum (Carbine et al., 2019, Xu et al., 2011). However, in our study, only UF showed obvious abnormalities in the OW group. These findings may suggest earlier demyelination in the UF than in other WM structures during the period of OW, in which the destruction of WM fiber bundles may begin in the OW stage and become more extensive during the progression of overweight to obesity. Our study also found that FA and ODI values of the right UF were associated with cognitive domains to varying degrees, especially in memory, which is consistent with the previous findings (Park et al., 2019).

In contrast, little literature focuses on the microstructure changes of the MCP and the PCT, and their association with RCFT‐D, language, and the cognition‐related plasma biomarkers of OW adults. Our findings seek to open a new research perspective for OW research. The MCP and PCT are the main afferent pathways to the cerebellum and are composed of white matter fibers originating from the contralateral pontine nuclei. The pontine nuclei are part of the corticopontocerebellar pathway that controls not only the action of motor tasks but also the planning and initiation of movements (Kuang et al., 2023). Thus, both walking and speaking could be affected (Morales and Tomsick, 2015), and the MCP and PCT injury may be associated with an increased risk of cognitive impairment (Hu et al., 2021, Dadar et al., 2022, Okugawa et al., 2005), which could be susceptible to the disruption of cerebellar access to motor, cognitive, and limbic afferent information from the cerebral cortex (Tobyne et al., 2017).

In addition, the damaged WM areas are also correlated with OW‐induced cognitive impairment. Although our study found no differences in cognitive function between groups, abnormal fiber bundles in the WM of the OW groups suggest underlying impairment in the cognitive microstructure and brain function of OW patients. Our study showed that the WM tracts, severely damaged in OW patients, were associated with clinical cognitive functions. UF is a white matter tract connecting the limbic system to the orbital frontal cortex (Von Der Heide et al., 2013) with a significant impact on memory, language, and emotional processing (Alm et al., 2016). Many studies have documented the association of MCP and PCT with memory, emotional, and language skills (Toescu et al., 2022, Kilroy et al., 2022, Jossinger et al., 2022). These also support that OW contributes to the occurrence of cognitive diseases. Further analysis showed that the damage to nerve fibers mainly occurred bilaterally; most of them were projection and long association fibers, which is consistent with previous studies (Carbine et al., 2019, Karlsson et al., 2013, Spangaro et al., 2018).

Although rarely has literature reported brain pathology in OW, the association between OW and WM microstructure abnormalities may be explained by several potential mechanisms. The first potential mechanism is neuronal injury. A previous study shows that sensorimotor control might be considered a key pathology in OW individuals (Li et al., 2022). The weakening in sensorimotor control was associated with MCP, PCT, and ICP, which were transmitting sensory and motor information from the cerebral cortex or spinal cord to the cerebellum and regulate the role of cerebellum function (Tobyne et al., 2017). These were confirmed in the OW of our study. We speculate that the damage to key fiber bundles may be sufficient to reduce the processing speed of information transmission caused by discontinuous connections between the information hemispheres of the brain (Han et al., 2013). In addition, the damage to WM above may also induce motor incoordination and lead to further weight gain. Higher body weight could further increase cognitive risk (Hu et al., 2022). The second potential mechanism is brain atrophy. Recent reports show that obesity is linked to Alzheimer's disease (AD)‐related brain volume loss, especially in the temporal and frontal lobes (Morys et al., 2023, Raji et al., 2023). This may be attributed to obesity‐related insulin resistance, leading to reduced glucose intake (Steiner et al., 2014). In our study, the UF is one of the most typically occurring WM injury regions in OW and has traditionally been considered as part of the temporal lobes (Von Der Heide et al., 2013), which have a close relation with brain atrophy and cognitive domain (Chapuis et al., 2013). The third potential mechanism is reward‐related. Our study also found decreased white matter integrity in the UF, which may be associated with reward‐related neural circuitry (Carbine et al., 2019). OW populations may have a heightened reward response to food. This could probably explain why the weight of some OW continued to increase. The fourth potential mechanisms were associated with insufficient blood supply. OW might cause hardening of the arteries (Candela et al., 2016), which could lead to the breakdown of the microstructural integrity of the WM (Zhai et al., 2016). The UF damage may impair blood pressure regulation (Cheng et al., 2024). All these pathogenic factors can further compromise the blood supply to the brain. The other possible mechanisms include cerebrovascular dysfunction, inflammation‐related pathologies, and dysfunction of microglia (Alhurani et al., 2016, Horvath et al., 2010). However, due to the lack of research on these aspects of OW, the exact pathogenic mechanism remains to be resolved.

Our study found that the levels of NfL and GFAP decreased in overweight individuals, as evidenced by similar findings in obese individuals (Rebelos et al., 2022). The significant reduction of NfL and GFAP in the overweight group may be related to individual metabolic characteristics or inflammatory levels (Neto et al., 2023). Previous studies had suggested that NfL and GFAP could reflect neurodegeneration, and are associated with various neurodegenerative diseases (Stocker et al., 2022), such as neuroinflammation (Kuhle et al., 2019), brain edema–induced diseases, traumatic brain injury (Shahim et al., 2024), AD (Oeckl et al., 2019), and mental disorders (Li et al., 2019). When neural/astrocytic damage occurs, NfL and GFAP are released in large amounts into the CSF and blood. In conflict with this, overweight individuals with neurodegeneration had lower levels of NfL and GFAP. There may be some reasons. First, the levels of NfL and GFAP in the blood were not stable, and they reflect the dynamic changes in neurodegeneration, metabolism, and inflammation. For example, Barba L. found that the levels of NfL and GFAP exhibit dynamic changes at different time points in patients with moderate‐to‐severe acute ischemic stroke, and these changes were closely related to treatment outcomes and functional recovery (Barba et al., 2023). Rebelos E. found that circulating levels of these proteins have been found to be lower compared to lean individuals, and they increase following weight loss induced by bariatric surgery (Rebelos et al., 2022). These suggest that the levels of NfL/GFAP in the blood are closely related to metabolic changes and different stages of neurodegeneration. Second, we speculate that the elevated levels of GFAP and NfL in the blood may reflect neuroaxonal or astrocytic damage in an active pathological state, such as TBI (Shahim et al., 2024) and MS (Kuhle et al., 2019). Despite neural/astrocytic damage in overweight individuals, they may be in an inactive state without progressive damage. Our results also found that there were no significant differences in cognitive function between the two groups, which suggests that overweight individuals had not experienced progressive neurodegeneration. Thus, NfL and GFAP are not released in large amounts. On the contrary, the decrease in the blood levels of NfL and GFAP at this time may be primarily regulated by metabolic characteristics or inflammatory levels.

The strengths of our investigation recruited subjects with complete clinical, neuropsychological, plasma biomarkers, and NODDI data. Hence, we were able to investigate the differences in WM microstructure between OW and SW, and the association of these differences with cognition and cognitive‐related biomarkers. In addition, we used an advanced NODDI model, which provides finer indicators of tissue microstructure than DTI.

Our study was not without limitations. First, subject to the relatively small sample size, we did not further analyze the impact of diseases such as diabetes, hyperlipidemia, and hypertension on cognitive function and microstructure. In subsequent research, we plan to incorporate a broader range of metabolic indicators to assess their potential impacts. Second, the results are derived from a Chinese population, and caution needs to be taken when they are generalized to other populations. Third, owing to its cross‐sectional design, selection bias was inevitable.

In conclusion, our study provides evidence that changes in the WM microstructure are broadly associated with a wide range of cognitive domains and cognition‐related plasma biomarkers and that UF, MCP, and PCT may be key targets for early intervention of OW. These findings suggest that the abnormalities of the WM microstructure may serve as early indicators of OW‐related cognitive dysfunction, and early intervention should be initiated to curb the progression of OW to obesity.

## Author Contributions

**Shaofan Jiang**: data curation, formal analysis, investigation, writing – original draft. **Yingzhe Cheng**: formal analysis, investigation, visualization, writing – original draft. **Rifeng Jiang**: investigation, formal analysis, visualization, writing – original draft. **Peilin Huang**: data curation. **Jiejun Zhang**: data curation. **Xiaodong Pan**: conceptualization, data curation, formal analysis, funding acquisition, investigation. **Yunjing Xue**: conceptualization, project administration, writing – review and editing.

## Ethics Statement

All procedures performed in studies involving human participants were following the ethical standards of the institutional research committee and with the 1964 Helsinki Declaration and its later amendments or comparable ethical standards. The study protocol was approved by the Ethics Committee of Fujian Medical University Union Hospital(2021KJT001).

## Consent

All participants provided written informed consent at the time of study registration and DWI data collection. Informed consent for participants with severe cognitive impairment was obtained from their guardians.

## Conflicts of Interest

The authors declare no conflicts of interest.

## Peer Review

The peer review history for this article is available at https://publons.com/publon/10.1002/brb3.70777

## Data Availability

The data that support the findings of this study are available from the corresponding author upon reasonable request.
